# Dietary sodium intake and all-cause mortality in kidney stone patients: a retrospective cohort study

**DOI:** 10.3389/fnut.2025.1623936

**Published:** 2025-06-19

**Authors:** Shuangning Liu, Yu Dai, Baolei Shi, YanHu Meng, Xiaoke Sun, Yatao Jia

**Affiliations:** ^1^Department of Urology, Baoji People's Hospital, Baoji, Shaanxi, China; ^2^Department of Urology, Honghui Hospital, Xi'an Jiaotong University, Xi'an, Shaanxi, China

**Keywords:** sodium, dietary, kidney stone, all-cause mortality, National Health and Nutrition Examination Survey

## Abstract

**Background:**

The long-term effects of dietary sodium intake on patients with kidney stones remain unclear; hence, this study aims to investigate the correlation between dietary sodium intake and all-cause mortality in patients with kidney stones.

**Methods:**

This retrospective cohort study included 2,765 participants from the National Health and Nutrition Examination Survey (NHANES) 2007–2018. The National Death Index (NDI) was utilized to identify all causes of death until December 31, 2018. Hazard ratios (HR) and 95% confidence intervals (CIs) were calculated using multivariate Cox regression models. Subsequently, subgroup analysis, Kaplan–Meier (KM) curves, as well as weighted generalized additive model regression and smooth curve fitting were performed to further evaluate the correlation between dietary sodium intake and all-cause mortality.

**Results:**

Over the 17,901 person-years of observation, a total of 372 deaths were recorded. The baseline characteristics revealed that individuals with elevated dietary sodium intake tended to be younger, Non-Hispanic White people, with a higher educational attainment, stable marital status, higher household income, lower prevalence of coronary heart disease (CHD), and were more inclined to smoking and alcohol consumption compared to participants with lower sodium intake (<2.2 g/d) (*p* < 0.01). In the fully adjusted Model 4, a significant inverse relationship between dietary sodium intake (DSI) and all-cause mortality risk was observed when DSI was analyzed as a continuous variable (HR = 0.89, 95% CI = 0.80–0.99, *p* = 0.034). When DSI was treated as a categorical variable, individuals with a DSI ≥ 2.2 g/day exhibited a reduced risk of all-cause mortality compared to the lowest sodium intake group (DSI < 2.2 g/d). The relationship between dietary sodium intake and all-cause mortality in kidney stone patients demonstrated a linear association, with an 11% decrease in the risk of all-cause mortality observed for each additional unit-g/d increase in dietary sodium intake.

**Conclusion:**

Higher dietary sodium intake levels were associated with lower all-cause mortality in kidney stone patients within the United States population. Notably, our results contradict the currently widely advocated recommendation to reduce sodium intake. Nonetheless, this observational study alone is insufficient to support any specific dietary recommendations.

## Introduction

Kidney stones are urological disorders resulting from the abnormal accumulation of crystalline substances such as calcium, oxalic acid, and uric acid in the kidneys (1). The pathogenesis of this condition is intricate, involving a complex interplay of genetic, metabolic, environmental, and dietary factors. Among these factors, diet is a modifiable exogenous element that significantly influences stone formation (2). Current research (3, 4) indicates that hypercalciuria, a fundamental metabolic risk factor for calcium stones, is found in 85% of kidney stone cases. The underlying pathological mechanisms encompass aberrant intestinal calcium absorption, impaired renal tubular calcium reabsorption, and disrupted bone calcium release. Notably, dietary sodium intake (DSI) directly enhances urinary calcium excretion by competitively inhibiting renal tubular calcium reabsorption (5–8); however, the relationship between DSI and kidney stone risk remains controversial. While previous studies have explored the short-term effects of DSI on urinary calcium metabolism, its long-term impact on the prognosis of kidney stone patients remains unclear. Moreover, large cohort studies investigating the relationship between DSI and the risk of all-cause mortality are lacking. Therefore, the present study analyzed data from the National Health and Nutrition Examination Survey (NHANES) spanning 2007–2018 to explore the independent correlation between DSI and all-cause mortality in individuals with kidney stones.

## Methods

### Data sources and study population

**Figure 1:**
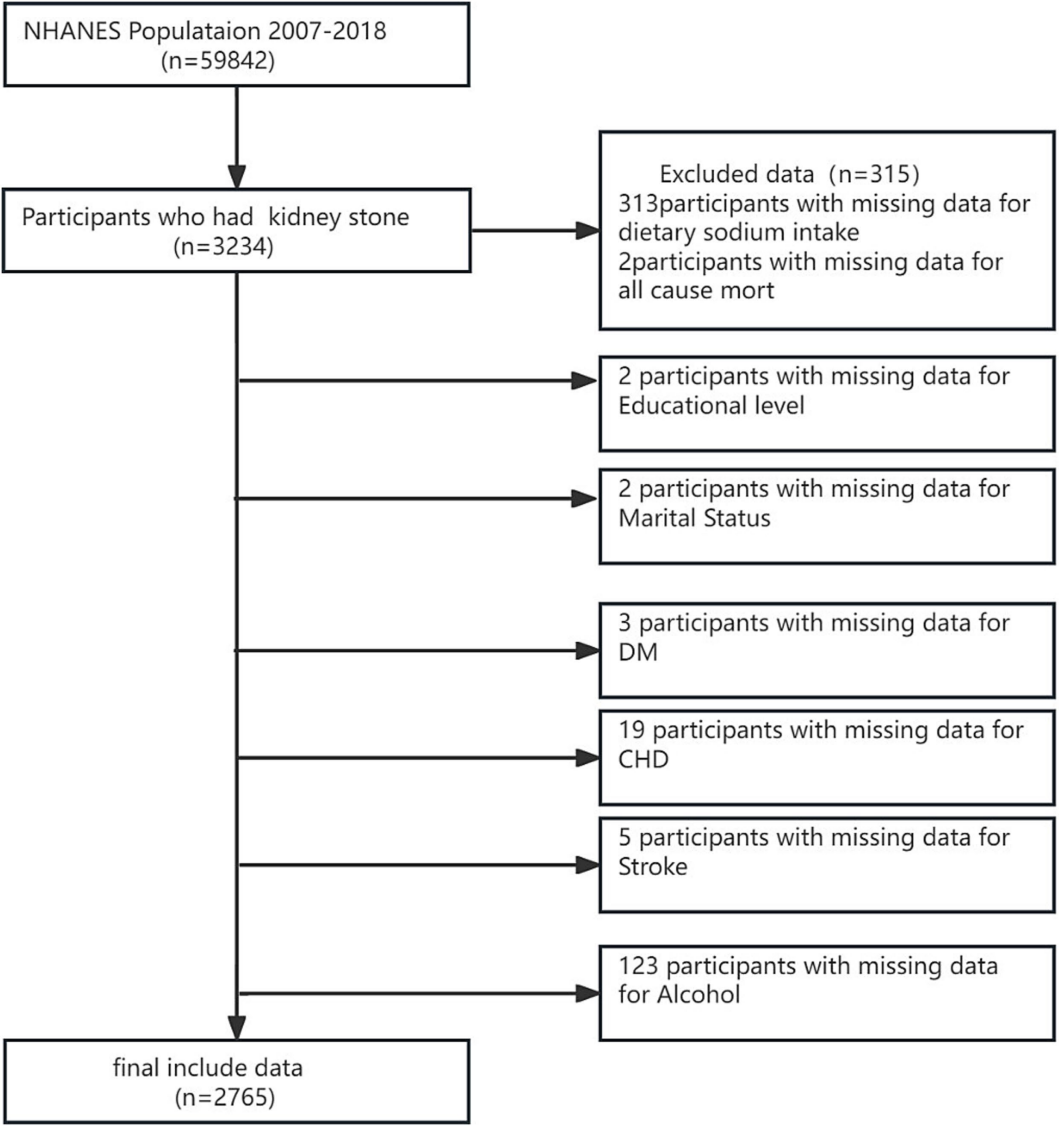
Flowchart of participant selection.

The National Health and Nutrition Examination Survey (NHANES) is a comprehensive national probability sample of the entire non-institutionalized civilian population in the USA, covering individuals aged 2 months and above. The survey collected a wide array of data, such as demographic information, socioeconomic status, dietary behaviors, and various health-related details, and involved thorough examinations and laboratory tests administered by highly skilled medical professionals. NHANES data are publicly accessible through the NHANES website.^1^ Our study utilized data from six NHANES cycles spanning 2007–2018. The surveys have been approved by the NCHS Institutional Review Board, and participants have provided informed written consent. The study focused on adults aged 20 years and above who completed the survey. Subjects without relevant data on kidney stones, mortality, or DSI were systematically excluded, resulting in a final sample of 2,765 subjects (Figure 1).

### Measurement of DSI

In NHANES, a 24-h recall was used to inquire about all foods and beverages consumed from midnight to midnight on the day preceding the interview. Nutrient data associated with the consumption were sourced from the United States Department of Agriculture’s Food and Nutrient Database for Dietary Studies. The average sodium intake (mg/day) was calculated using dietary recalls from each survey period. The recommended sodium intake was defined as being below 2,300 mg/day. Referring to *the Dietary Guidelines for Americans 2020–2025* (9), DSI was categorized into four groups (<2.2 g/d, 2.2–3.1 g/d, 3.1–4.2 g/d, >4.2 g/d).

### Ascertainment of kidney stones

Data on the prevalence of kidney stone disease were obtained from the interview records. Participants were asked, “Have you ever experienced a kidney stone?” during the standardized home interview. Adult participants who answered affirmatively were categorized as having a history of kidney stones.

### Ascertainment of mortality

Data on mortality status and follow-up duration (in months) were collected from death certificates in the National Death Index (NDI), covering the period from survey participation to December 31, 2018. Our study’s primary findings focused on mortality from all causes.

### Assessment of covariates

Demographic and socioeconomic data, such as age, gender, race/ethnicity, education level, marital status, poverty income ratio (PIR), and smoking status, were collected through structured household interviews using a standardized questionnaire. In addition, height, weight, and alcohol consumption were measured during the participants’ visits to mobile health screening centers for medical assessments. Body mass index (BMI) was computed according to a standardized procedure by dividing the weight in kilograms by the square of height in meters (10). Race/ethnicity categories comprised Mexican American, Non-Hispanic White, Non-Hispanic Black, and other races. Education levels were classified as less than high school, high school diploma or equivalent, and college education or higher. Marital status was categorized as ‘married’ and ‘not married’, encompassing individuals who had never been married, those living with a partner, and others. Family income was determined based on the poverty income ratio (PIR), which considered specific thresholds according to family size (≤1.3, 1.3–3.5, >3.5). Smoking status was classified into three groups: “never smoker” for those who had never smoked cigarettes, “former smoker” for individuals who had smoked ≥100 cigarettes in the past but had quit, and “current smoker” for those who had previously smoked ≥100 cigarettes and were still smoking at the time of the interview (11). Drinking status was determined by whether the individual consumed 12 or more alcoholic beverages per year (12). A self-reported medical history of diabetes, hypertension, cardiovascular disease, and stroke was also collected.

### Statistical analysis

Histogram distribution, Q–Q plot, or the Kolmogorov–Smirnov test was used to determine whether variables conformed to a normal distribution. All normally distributed continuous variables were presented as mean ± SD, while skewed continuous variables were described as median (interquartile range [IQR]). Categorical variables were presented as frequencies (%). The chi-square (categorical variables), One-Way ANOVA test (normal distribution), or Kruskal-Wallis H test (skewed distribution) was employed to analyze differences among different DSI groups.

Hazard ratios (HRs) and 95% CIs were calculated to determine the association between all-cause mortality and DSI by using Cox proportional hazards models. Participants who were lost to follow-up were censored at that particular time point. The outcome of all-cause mortality was assessed with Kaplan–Meier survival curves according to the DSI (as a categorical variable) and evaluated with the log-rank test. Confounders were selected based on their associations with the outcomes of interest, or a change in the effect estimate of more than 10%. Specifically, four models were constructed: (1) unadjusted; (2) adjusted for age and gender; (3) adjusted for age, gender, and race; and (4) adjusted for age, gender, race, BMI, hypertension, diabetes mellitus, coronary heart disease, stroke, smoking, and alcohol consumption. Subgroup analyses were performed to assess the link between DSI and all-cause mortality, with interaction *p*-values tested within each subgroup. Weighted generalized additive model regression and smoothed curve fitting were employed to delve deeper into the association between DSI and all-cause mortality. The calculated effect sizes and *p* values from all these models were reported and compared. All analyses were performed using R Statistical Software (Version 4.2.2, The R Foundation)^2^ and Free Statistics analysis platform (Version 1.9, Beijing, China).^3^ FreeStatistics is a software package that provides intuitive interfaces for most common analyses and data visualization. The software uses R as the underlying statistical engine, and the graphical user interface (GUI) is written in Python. This package is designed for reproducible analysis and interactive computing. In this study, a two-sided *p* value < 0.05 was considered statistically significant.

## Results

### Baseline characteristics of participants

Among the 2,765 adults diagnosed with kidney stone disease, the median age was 56.1 years (25th percentile: 48 years, 75th percentile: 64 years), with 55.57% being male. Within this cohort, 25.0% had a low DSI (<2.2 g/day). Table 1 presents the weighted sociodemographic characteristics and various disease conditions across the four groups. Individuals with higher DSI tended to be younger, with a higher proportion of Non-Hispanic White people, higher educational attainment, stable marital status, higher household income, lower prevalence of coronary heart disease (CHD), and higher likelihood of smoking and alcohol consumption compared to participants with lower sodium intake (<2.2 g/d) (*p* < 0.01). However, no significant difference in body mass index (BMI), hypertension, diabetes mellitus (DM), and stroke was found among the four groups (all *p value > 0.05*).

**Table 1:** Baseline characteristics of kidney stone participants in NHANES data from 2007 to 2018, weighted.

Variables	Total	DSI* (g/d)				*p* value
		<2.2	2.2–3.1	3.1–4.2	>4.2	
Number^a^	2,765	691 (25.0)	690	691	693	
Gender, *n* (%)						<0.001
Male	1,555 (55.57)	292 (38.80)	345 (45.74)	408 (58.84)	510 (75.12)	
Female	1,210 (44.43)	399 (61.20)	345 (54.26)	283 (41.16)	183 (24.88)	
Age, years, Mean (±SD)	56.1 ± 16.1	58.1 ± 16.2	57.9 ± 15.6	56.4 ± 16.1	52.2 ± 15.6	<0.001
BMI, kg/m^2^, Mean (±SD)	30.6 ± 6.9	30.1 ± 6.4	30.4 ± 6.8	31.1 ± 7.4	30.8 ± 6.9	0.066
Race, *n* (%)						0.038
Non-Hispanic White	1,523 (76.72)	347 (74.46)	378 (75.80)	403 (79.85)	395 (76.49)	
Non-Hispanic Black	369 (5.88)	99 (6.77)	92 (5.84)	89 (5.22)	89 (5.79)	
Mexican American	354 (6.47)	100 (6.99)	81 (5.78)	73 (4.74)	100 (8.29)	
Others	519 (10.93)	145 (11.79)	139 (12.59)	126 (10.19)	109 (9.43)	
Educational level, *n* (%)						<0.001
<High school	277 (4.37)	111 (7.70)	73 (4.25)	53 (3.13)	40 (2.87)	
High school	1,017 (33.58)	258 (34.03)	262 (36.21)	224 (28.99)	273 (35.19)	
>High school	1,471 (62.05)	322 (58.27)	355 (59.54)	414 (67.89)	380 (61.94)	
Marital Status, *n* (%)						<0.001
Married or living with partner	1,769 (68.30)	386 (59.53)	443 (68.57)	473 (73.42)	467 (70.54)	
Living alone	996 (31.70)	305 (40.47)	247 (31.43)	218 (26.58)	226 (29.46)	
PIR, *n* (%)						<0.001
≤1.3	784 (18.82)	245 (25.05)	190 (18.37)	174 (17.58)	175 (15.21)	
1.3–3.5	994 (35.05)	229 (36.83)	249 (34.56)	263 (34.74)	253 (34.32)	
>3.5	987 (46.13)	217 (38.12)	251 (47.07)	254 (47.68)	265 (50.48)	
Hypertension, *n* (%)						0.225
No	1,564 (59.08)	388 (60.16)	381 (56.69)	380 (57.60)	415 (61.68)	
Yes	1,201 (40.92)	303 (39.84)	309 (43.31)	311 (42.40)	278 (38.32)	
DM, *n* (%)						0.473
No	2,136 (81.29)	529 (79.91)	525 (78.85)	532 (81.32)	550 (84.57)	
Yes	629 (18.71)	162 (20.09)	165 (21.15)	159 (18.68)	143 (15.43)	
CHD, *n* (%)						0.05
No	2,537 (93.37)	629 (92.79)	622 (91.81)	634 (93.74)	652 (94.90)	
Yes	228 (6.63)	62 (7.21)	68 (8.19)	57 (6.26)	41 (5.10)	
Stroke, *n* (%)						0.451
No	2,597 (95.11)	644 (93.25)	643 (95.02)	653 (95.94)	657 (95.97)	
Yes	168 (4.89)	47 (6.75)	47 (4.98)	38 (4.06)	36 (4.03)	
Smoking, *n* (%)						0.01
Never	1,350 (49.61)	344 (47.87)	375 (53.84)	318 (49.48)	313 (47.41)	
Former	874 (31.23)	203 (28.91)	199 (30.07)	237 (32.63)	235 (32.87)	
Current	541 (19.16)	144 (23.22)	116 (16.08)	136 (17.90)	145 (19.72)	
Alcohol, *n* (%)						<0.001
No	709 (20.35)	230 (24.79)	186 (24.39)	163 (17.17)	130 (16.08)	
Yes	2,056 (79.65)	461 (75.21)	504 (75.61)	528 (82.83)	563 (83.92)	

DSI dietary sodium intake, BMI Body mass index, PIR poverty income ratio, DM diabetes, CHD Coronary Heart Disease.

Other: other than Non-Hispanic White, Non-Hispanic Black, and Mexican American, including multiracial.

^a^Data are presented as unweighted number (weighted percentage) for categorical variables and mean (standard error) for continuous variables.

*Dietary sodium intake was entered as a continuous variable (per 1,000 unit).

**Figure 2:**
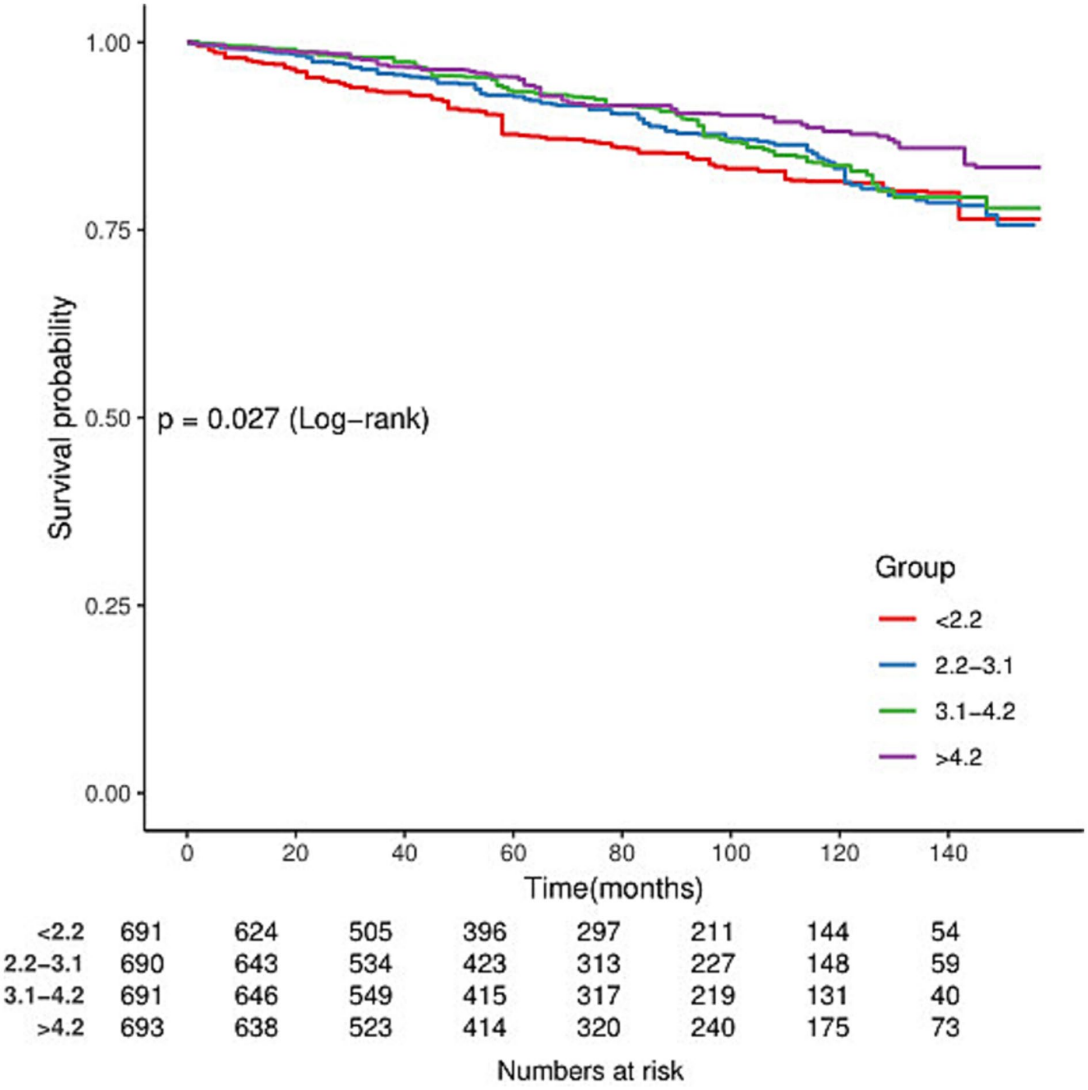
Kaplan–Meier survival curves for all-cause mortality by dietary sodium intake (DSI) in adults with kidney stones.

**Figure 3:**
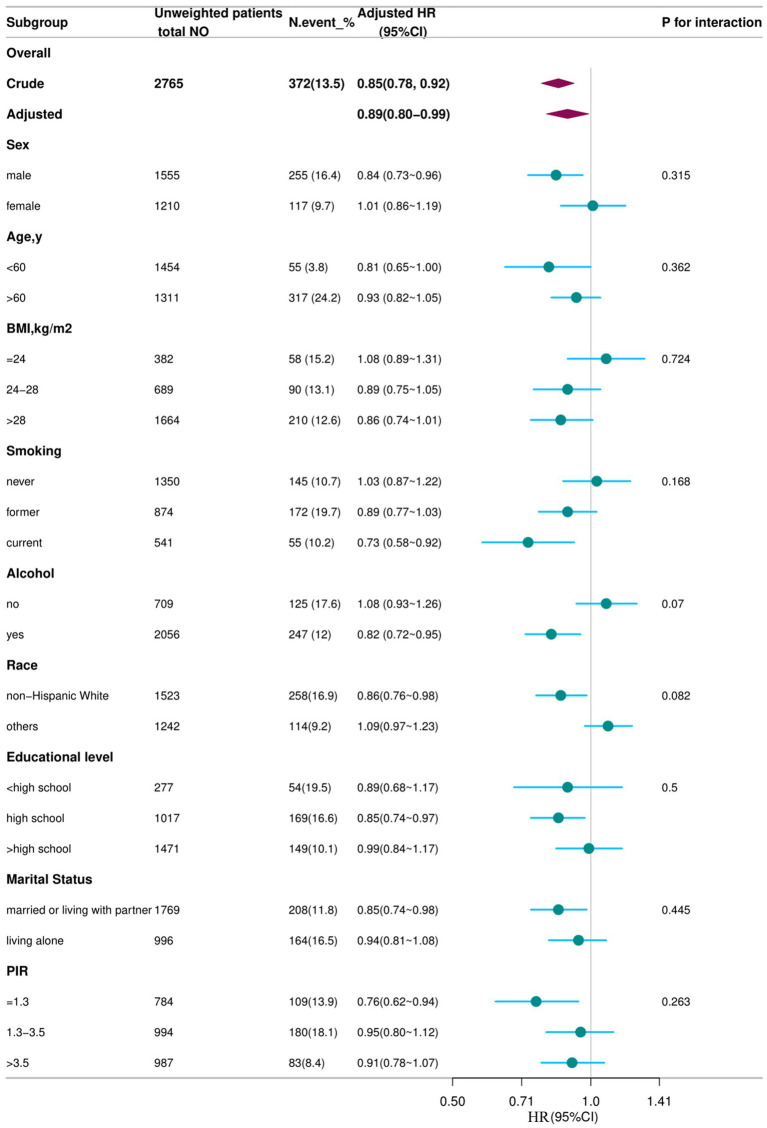
Association between DSI and all-cause mortality according to general characteristics. Each subgroup was adjusted for all variables (sex, age, BMI, smoking status, alcohol drinking status, race/ethnicity, education level, marital status, family poverty income, and NHANES cycle) except for the stratification factor itself. Circles represent the HRs, and horizontal lines represent 95% CIs. Diamonds represent the overall HR, with the outer points of the diamonds representing the 95% CI. BMI, body mass index; PIR, family poverty income; CI, confidence interval; HR, hazard ratio; NHANES, National Health and Nutrition Examination Survey.

**Figure 4:**
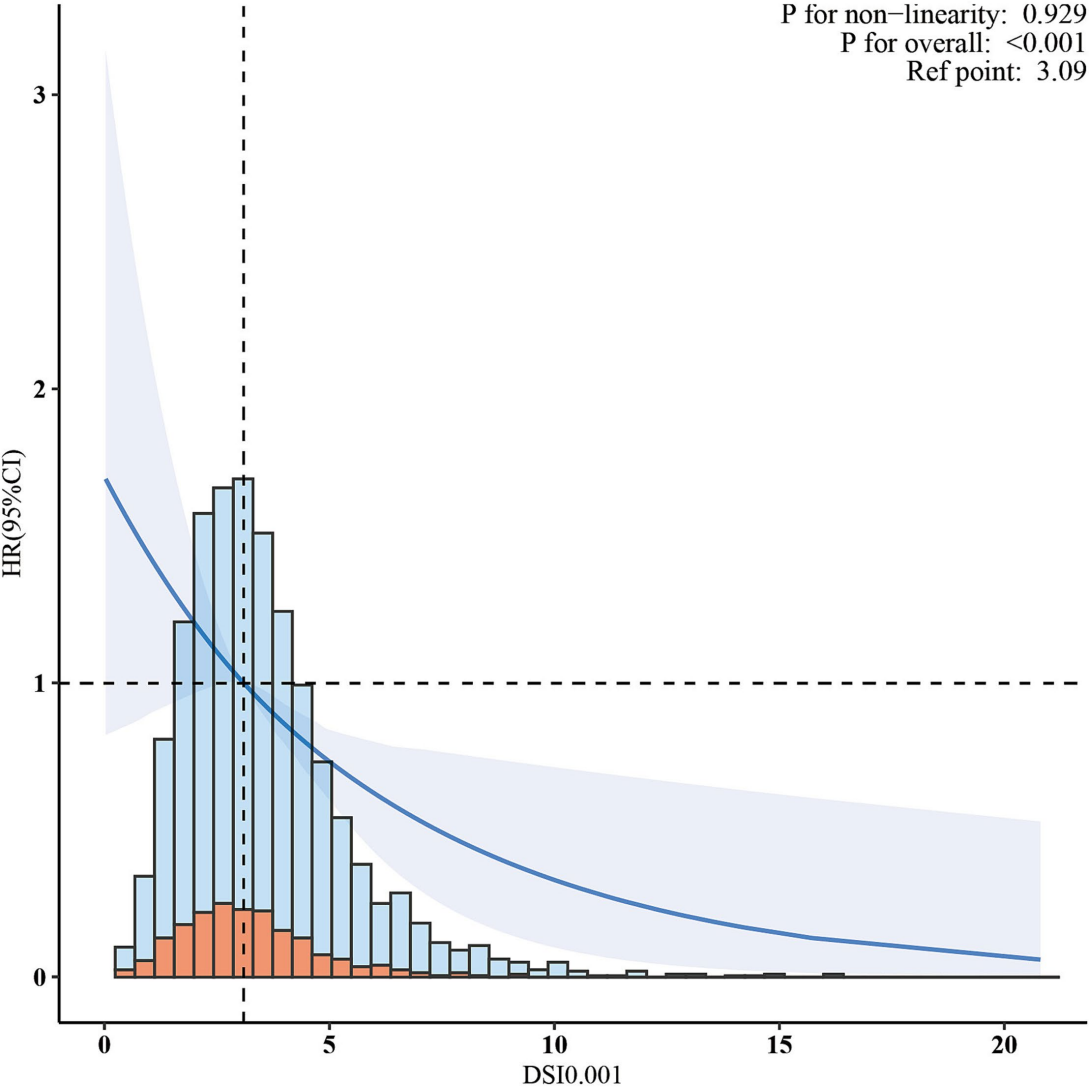
The association between dietary sodium intake and all-cause mortality. The solid line and shaded area represent the predicted value and 95% confidence interval, respectively.

In the cohort of 2,765 patients, all-cause mortality showed an incidence rate of 13.5%. Univariate analysis revealed a significant inverse association between DSI and mortality, as evidenced by the Kaplan–Meier curves (log-rank test *p* = 0.027, Figure 2). The multivariable Cox proportional hazards analysis (Table 2) indicated that higher sodium intake was associated with a reduced mortality risk (per 1,000 units: HR = 0.89, 95% CI = 0.80–0.99, *p* = 0.034) in model 4, which was adjusted for confounders. The results suggested an 11% decrease in the risk of all-cause mortality for each additional g/day increase in DSI. Adjusted hazard ratios (95% CIs) for mortality in sodium intake groups 2, 3, and 4 (2.2–3.1 g/d, 3.1–4.2 g/d, >4.2 g/d) were 0.78 (95% CI = 0.54, 1.13), 0.66 (95% CI = 0.46, 0.96), and 0.70 (95% CI = 0.44, 1.13) compared to group 1 (<2.2 g/d) (Table 2, model 4). The model shows that participants in groups 2, 3, and 4 had a 22, 34, and 30% reduction in mortality incidence, respectively, compared to group 1 (Table 2, model 4). As depicted in Figure 3, the interaction between DSI and all-cause mortality was analyzed in participants subgroup by age (<60 or ≥60 years), gender (female or male), BMI (<24, 24–28, or ≥28 kg/m^2^), smoking status (never smoker, former smoker, or current smoker), drinking status (no or yes), race (Non-Hispanic White or others), education level (<high school, high school, >high school), and PIR (<1.3, 1.3–3.5, >3.5). No significant interactions were observed between DSI and other stratified variables (*p for interaction >0.05*). Figure 4 depicts the dose–response correlation between DSI and all-cause mortality. Following comprehensive adjustments, a linear correlation was observed (*p for nonlinearity > 0.05*). Each increase of 1,000 units (g/d) in DSI correlated with an 11% decrease in the risk of all-cause mortality (refer to Table 2).

**Table 2:** Hazard ratios for all-cause mortality associated with dietary sodium intake in adults with kidney stones from NHANES 2007–2018.

Variable	DSI		DSI levels quartile (g/day)			
			<2.2 (*n* = 691)	2.2–3.1 (*n* = 690)	3.1–4.2 (*n* = 691)	>4.2 (*n* = 693)
	HR (95%CI)	*p* value	HR (95%CI)	HR (95%CI)	HR (95%CI)	HR (95%CI)
**All-cause mortality**						
**Number of deaths/total**	372/2,765		105/691	100/690	97/691	70/693
Model 1	0.85 (0.78, 0.92)	<0.001	1.00 (ref)	0.78 (0.54, 1.13)	0.71 (0.48, 1.04)	0.56 (0.38, 0.81)
Model 2	0.89 (0.80–0.99)	0.032	1.00 (ref)	0.84 (0.57, 1.23)	0.68 (0.47, 1.00)	0.72 (0.46, 1.14)
Model 3	0.89 (0.80–0.99)	0.034	1.00 (ref)	0.84 (0.57, 1.23)	0.68 (0.46, 1.01)	0.72 (0.46, 1.14)
Model 4	0.89 (0.80–0.99)	0.034	1.00 (ref)	0.78 (0.54, 1.13)	0.66 (0.46, 0.96)	0.70 (0.44, 1.13)

DSI, dietary sodium intake.

Model 1: Unadjusted.

Model 2: Adjusted for NHANES cycle, age, gender.

Model 3: Adjusted for Model 2 + race.

Model 4: Adjusted for Model 3 + BMI, hypertension, DM, CHD, stroke, smoking, alcohol.

## Discussion

In this extensive cohort study of the US population with kidney stones, a novel negative correlation was found between DSI and the risk of all-cause mortality. This association was consistently observed across various clinical subgroups and sensitivity analyses, highlighting its clinical significance.

Earlier studies investigating the relationship between DSI and all-cause mortality have reported conflicting results. For instance, a previous meta-analysis (13) and a multicenter study (14) found no significant association between DSI and all-cause mortality. In contrast, another meta-analysis performed in 2014 revealed a U-shaped association between DSI and all-cause mortality (15), indicating that both low sodium intake and high sodium intake were associated with increased mortality.

However, several other studies reported controversial findings. Cohen et al. (16) and Stolarz-Skrzypek et al. (17) have demonstrated a negative relationship between sodium intake and overall mortality. Similarly, our study observed an inverse correlation within a US population, specifically focusing on individuals with kidney stones. This conclusion was further supported by other stratified analyses, including subgroups according to age, sex, BMI, smoking status, alcohol drinking status, race/ethnicity, education level, marital status, and family poverty income. The variability in findings across these studies may be partially attributed to variations in study cohorts, sample sizes, and the consideration of potential confounding variables, necessitating further investigation for validation. DSI plays a key role in maintaining normal blood volume and blood pressure levels; although reducing sodium intake can lower blood pressure in people with hypertension, excessive sodium restriction may have adverse effects in the general population, especially in people with kidney stones. Insufficient sodium intake may lead to decreased blood volume (18). In addition, a low-sodium diet may activate the renin-angiotensin system, further affecting blood pressure regulation (19). Moreover, hypotension may exert adverse effects on blood perfusion to vital organs, especially in the cardiovascular system. Studies have shown that low diastolic blood pressure (less than 60 mmHg) is significantly associated with increased all-cause mortality, especially in patients with pre-existing cardiovascular disease (20). Moderate sodium intake is also essential for maintaining electrolyte balance in the kidneys (21). Electrolyte disorders may have adverse effects on the heart, neuromuscular, and other functions, and even endanger life in severe cases. For patients with kidney stones, preserving renal function is a major factor in preventing stone recurrence and avoiding renal impairment, thereby indirectly reducing the risk of all-cause mortality (22). Some studies suggest a possible relationship between sodium intake and insulin sensitivity. Moderate sodium intake may help maintain normal insulin sensitivity and facilitate glucose metabolism and utilization (23, 24). In kidney stone patients, normal insulin sensitivity can prevent blood glucose metabolism disorders, reduce the risk of metabolic diseases such as diabetes, and exert a positive impact on all-cause mortality. Furthermore, moderate sodium intake can enhance appetite, allowing patients to eat more food and ensure adequate energy and nutrient supply (25). Good nutritional status is crucial for maintaining physical function, promoting disease recovery, and improving quality of life. Patients receiving adequate nutritional support also benefit from good resistance and immunity, thus reducing the risk of death from various diseases. Additionally, sodium may exert a certain antioxidant effect, which can help clear free radicals in the body and reduce oxidative stress damage. Oxidative stress is an important factor leading to a variety of chronic diseases and aging. Hence, reducing oxidative stress by higher sodium intake may have a positive impact on the overall health of patients with kidney stones, thereby reducing all-cause mortality (26). Moderate sodium intake may assist in regulating inflammation in the body. Excessive inflammatory response is closely related to the occurrence and development of many diseases, such as cardiovascular diseases, diabetes mellitus, tumors, etc. (21, 27, 28). Appropriate sodium intake may participate in controlling inflammation, thereby reducing the risk of related diseases in kidney stone patients, demonstrating a potential protective effect on all-cause mortality.

This study is the first to examine the correlation between DSI and all-cause mortality in a nationally representative cohort of kidney stone patients, analyzing comprehensive NHANES data to control for various potential confounders. Nevertheless, the limitations of the present study should be acknowledged. Firstly, dietary intake estimates may not reflect participants’ actual dietary intake, underestimating the intakes of some minerals, including sodium. In contrast, urinary sodium measurements can provide sufficiently acceptable population estimates. On the other hand, kidney stone cases were self-reported, and some participants may not be aware of having kidney stones, potentially leading to misclassification. Additionally, no information was collected regarding stone composition. Also, although we adjusted for multiple known confounders, residual confounding due to unmeasured lifestyle factors or health behaviors cannot be entirely excluded. The observed inverse association between sodium intake and mortality may reflect, in part, the influence of healthier lifestyles or socioeconomic status among individuals with higher sodium intake. Finally, our data remain observational, which are susceptible to confounding due to unmeasured factors or factors that are difficult to quantify. For example, the variability in DSI may be related to dietary composition, processing, and preparation; these differences may affect outcomes in individuals with kidney stones. Our cohort consisted solely of patients from the United States. Therefore, studies in other populations are needed to validate our results. Considering these limitations, rigorously designed multicenter randomized controlled trials (RCTs) are required to confirm our findings. This observational study will not override existing guidelines. Future efforts should focus on conducting additional randomized controlled trials.

## Conclusion

This retrospective cohort study found that elevated Dietary Sodium Intake (DSI) correlates with a reduced risk of all-cause mortality in individuals with kidney stones. These observational findings do not warrant specific dietary recommendations. Notably, they do not substantiate the prevailing advice for kidney stone patients to lower sodium intake.

## Data Availability

The raw data supporting the conclusions of this article will be made available by the authors without undue reservation.

## Footnotes

^1^ http://www.cdc.gov/nchs/-nhanes.htm

^2^ http://www.R-project.org

^3^ http://www.clinicalscient-ists.cn/freestatistics
